# Hydroclimate controls Congo peatland net oxygen release over the past 10,600 years

**DOI:** 10.1038/s41561-026-02031-z

**Published:** 2026-07-10

**Authors:** M. E. Galvez, S. Wu, Y. Garcin, E. Schefuß, G. Gassier, J. Lebamba, C. K. Kiahtipes, F. Bokomba Bwamangele, R. Kidebua Lutonadio, H.-P. Wotzka, T. Adatte, S. L. Jaccard

**Affiliations:** 1 University of Lausanne, Institute of Earth Sciences (ISTE), Lausanne, Switzerland; 2 Aix Marseille University, CNRS, IRD, INRAE, CEREGE, Aix-en-Provence, France; 3 MARUM - Center for Marine Environmental Sciences, University of Bremen, Bremen, Germany; 4 Institute of Prehistoric Archaeology, University of Cologne, Köln, Germany and Département de Biologie, Université des Sciences et Techniques de Masuku, Franceville, Gabon; 5 Institute for the Advanced Study of Culture and the Environment, University of South Florida, Tampa, FL USA; 6 Institut des Musées Nationaux du Congo (IMNC), Kinshasa, Democratic Republic of the Congo; 7 Institut für Ur- und Frühgeschichte, Forschungsstelle Afrika, Universität zu Köln, Cologne, Germany; 8 Present Address: State Key Laboratory of Deep-Sea Science and Intelligence Technology, Institute of Deep-Sea Science and Engineering, Chinese Academy of Science, Sanya, China

**Keywords:** Element cycles, Geochemistry

## Abstract

The burial of organic matter in marine environments is considered the dominant long-term source of atmospheric oxygen, yet the contribution of terrestrial burial remains poorly constrained. Quantifying any oxygen surplus associated with organic matter burial conventionally assumes a 1:1 oxygen-to-carbon stoichiometry. Here we aim to test this assumption and quantify net oxygen release from a terrestrial setting over time. We applied direct redox titration to a 10,600-year Congo Basin peat core, a hotspot of carbon accumulation. We find systematic deviation from the 1:1 ratio, enabling precise, time-resolved quantification of the net redox imbalance. Fluxes were strikingly nonlinear: drier phases triggered abrupt ~80% declines in net oxygen surplus within centuries, followed by rapid recovery when wetter conditions returned, revealing a hydroclimate-sensitive carbon–oxygen valve. Cumulative Holocene oxygen surplus reached 83 [68–100] petagrams of oxygen equivalents. Using the Congo oxygen-to-carbon ratio, we estimate that global peatland net oxygen production is commensurate with continental weathering oxygen sinks in the Holocene—a flux comparable in magnitude to marine burial but responding on orders-of-magnitude-shorter timescales. This finding provides qualitative insight into the contribution of terrestrial environments to Earth’s redox balance. It also offers a direct stoichiometric framework—applicable to coal archives—for quantifying that contribution across the Phanerozoic, independent of isotopic proxies, complementing existing marine-based models.

## Abstract (web summary)

The oxygen surplus from carbon burial in Congo peatlands reached 83 petagrams of net atmospheric oxygen equivalents during the Holocene, suggesting global peatlands may have offset 40–95% of continental weathering oxygen sinks, according to a peat core.

## Main

Earth’s oxygen cycle operates as a near-balanced biological loop: photosynthetic organisms fix CO_2_ into organic matter (OM) while releasing O_2_, and heterotrophic respiration largely reverses this process. Globally, this cycle turns over ~100 Pg carbon annually, with the fixation of one mole of carbon typically producing ~1 mole of O_2_ (ref. ^1^) (O_2_:C = 1) according to:

1 $${{\rm{CO}}}_{2}+{{\rm{H}}}_{2}{\rm{O}}={{\rm{CH}}}_{2}{\rm{O}}({\rm{OM}})+{{\rm{O}}}_{2}$$

Through this mechanism, an ecosystem such as the Amazon rainforest generates an annual O_2_ surplus of 20–40 Tg (Supplementary Section 9 and ref. ^2^), which is gradually offset over centuries by soil degradation and respiration^3^. Only the slight imbalance in this loop, the ~0.1 % of organic carbon that escapes respiration through sediment burial^4^ (equation (1)), results in a net liberation of oxygen to the atmosphere. Over geological timescales, this residual flux sustains atmospheric O_2_ and fuels the oxidative weathering of continental surfaces and oceanic crust^5^.

Models of the long-term carbon–oxygen cycle traditionally assign the dominant role in organic matter burial, and hence net O_2_ production, to marine deltaic and shelf sediments^6,7^. However, it has long been recognized that this marine-centric framework may overlook the importance of waterlogged terrestrial reservoirs^8^, in particular, peatlands. Despite covering just 3–5% of the present-day land surface today, peatlands sequester ~600 Pg (Table 1) of carbon accumulated during the Holocene^9–13^, a stock comparable in magnitude to the ocean’s dissolved organic C pool (~650 Pg (ref. ^14^)). By inhibiting organic matter decomposition under anoxic conditions, these ecosystems function in a manner analogous to marine sediments, effectively shielding carbon from oxidation.

**Table 1:** Holocene carbon accumulation and associated net O_2_ equivalents in tropical and boreal peatlands, with emphasis on the Congo Basin

Peatland region	Area (×10^3 ^km^2^)	Holocene C accumulation (Pg C)	Annual net O_2_ eq production (Tg O_2_ eq yr^−^^1^)	Cumulative net O_2_ eq production (Pg O_2_ eq)	Source (Area and C accumulation)
Localities					
Southeast Asian	200–450	68 [57–82]	16 [13–20]	190 [155–235]	refs. ^45–47^
Amazonia (Peru/Ecuador)	80–120	15 [10–20]	3.6 [2.4–4.8]	42 [28–56]	refs. ^48^^,49^
Congo Basin	145–186	30 [28–32]	7.2 [6.2–8.2]	84 [73–96]	refs. ^22,23^; this study
Aggregates					
Tropical peatlands	475–756	113 [84–134]	29.2 [22–33]	316 [256–387]	(see above)
Boreal and northern peatlands	3,100–3,900	480 [447–560]	109 [93–125]	1,280 [1,090–1,460]	refs. ^9,40,50^
Grand total	~4,000	593 [542–694]	135 [115–158]	1,596 [1,346–1,847]	refs. ^9,40,50^

Notes: Area and carbon accumulation values are midpoints compiled from the literature, with uncertainty ranges shown in brackets (see Sources). Net O_2_ eq production is then calculated by multiplying the Holocene carbon accumulation rate by the measured O_2_:C stoichiometry of 0.9–1.15 mol mol^−1^ (mean 1.025) from the Congo Basin IMB100 core, assumed here to be broadly representative of peat. Ranges reflect propagation of uncertainties in carbon stocks, peatland areas, and O_2_:C values. Tropical aggregates include additional smaller peatland areas not listed individually but reviewed in ref. ^45^.

Although the role of peatlands in the carbon cycle is well established, their influence on the long-term carbon–oxygen cycle remains largely unconstrained. Progress in quantifying this terrestrial contribution has been hindered by three main obstacles: (1) the scarcity of thick and continuous peat archives extending beyond the Last Glacial Maximum^10^ despite the widespread preservation of analogous deposits as coal throughout the Phanerozoic^15^; (2) conventional C and O isotopic proxies (that is, δ^13^C- and δ^18^O) struggle to unambiguously separate marine from terrestrial redox signals^16^ and (3) the reliance of most global models on fixed O_2_:C ratios, theoretical (1:1; equation (1)) or biosphere-averaged (~1.04–1.06) derived from short-term atmospheric O_2_/CO_2_ budgets or soil incubation experiments^17,18^, which obscure millennial-scale shifts in organic matter composition and decomposition state. Here, we overcome these limitations using a redox-titration method^19^ that directly quantifies the oxygen demand^20^ of preserved peat through high-resolution combustion experiments. This approach captures time-resolved diagenetic changes in lignocellulosic compound preservation, a signal that is averaged out in large-scale isotopic reconstructions (Methods and Supplementary Sections 1–4).

**Fig. 1:**
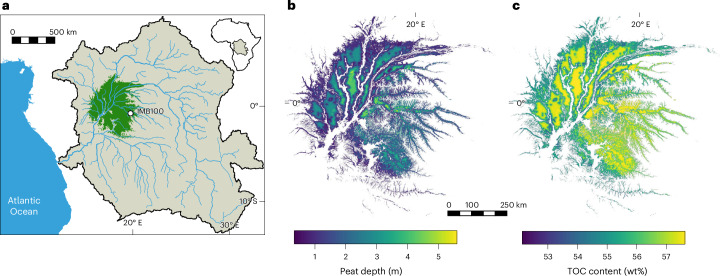
Overview of the sampling site in the central Congo Basin. **a**, Location of the central Congo peatlands, in green, the perimeter of the Congo Basin (black line) and the location of Imbonga (IMB100) peat core. **b**, Estimated peat depth map^23^. **c**, Total organic carbon (TOC) content (wt%) map, this study, interpolated from basin-wide field measurements^23^. Panel **a** adapted from ref. ^24^ under a Creative Commons license CC BY 4.0.

The Congo Basin, sometimes referred to as Earth’s ‘Second Green Lung’^21^, contains an extensive Holocene peat archive^22^ offering a unique window to examine long-term C accumulation in tropical terrestrial settings (Fig. 1). Whereas regional carbon stocks and isotopic compositions are well established^22–25^, the corresponding O_2_ dynamics have so far remained unconstrained. By combining high-resolution redox measurements of the IMB100 peat core^26^ with geospatial analysis (Methods), we show that the Congo peatland ecosystem has been persistently imbalanced towards net C burial, sustaining a long-term net O_2_ surplus throughout the Holocene. The intermittent character of the fluxes, however, provides empirical evidence for a slow–fast dynamic behaviour regulated by hydroclimatic and biogeochemical thresholds. These findings offer new mechanistic insights into the terrestrial control of Earth’s carbon–oxygen balance on millennial timescales.

## Peat properties and O_2_:C ratio

**Fig. 2:**
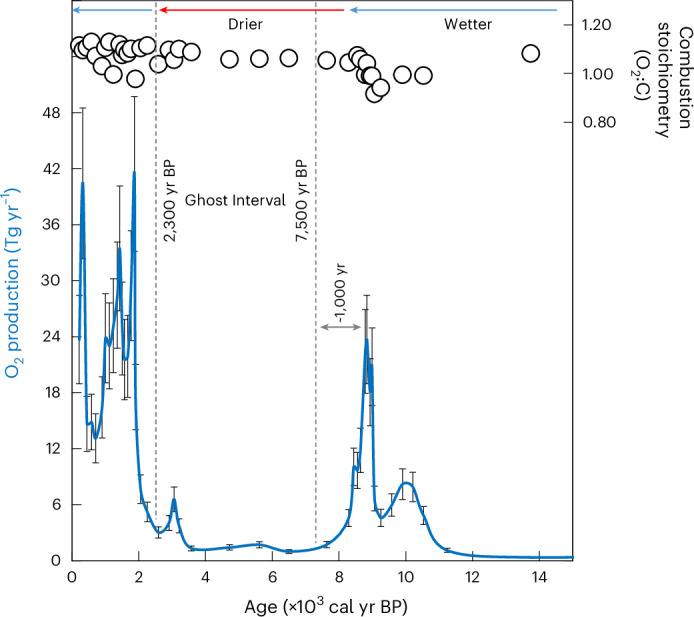
Oxygen demand and reconstructed O_2_ flux in Congo peatland. Black circles represent mean O_2_:C stoichiometry (0.9–1.15 mol mol^−1^) across climatic phases (right ordinate axis). The blue curve shows mean reconstructed O_2_ flux (left ordinate axis), with error bars representing the ±2 standard deviations (2σ) calculated by Monte Carlo simulation. The vertical dotted lines bracket the Ghost Interval (7.5–2.3 kyr before present (BP)). The O_2_:C ratio remains stable across both preserved and highly degraded intervals, whereas O_2_ fluxes decline by ~80% during drier phases and recover during wetter phases. Horizontal axis refers to calibrated years (×10^3^) before present (cal yr BP).

Peat from the IMB100 core (central Congo Basin; Methods and Supplementary Sections 1–4) is dominated by lignocellulosic OM, comprising both thermally labile and refractory organic fractions, with little relative changes in thermal reactivity throughout the sequence (Supplementary Section 4 and Supplementary Table 1). Total organic carbon contents remain relatively uniform (50 and 60 wt%), and the oxygen demand during combustion is consistently high over the last 10.6 kyr (Supplementary Sections 1 and 4). Remarkably, the derived combustion ratios (Supplementary Section 2) deviate from the canonical 1:1 value (equation (1)), yet fall within a narrow yet persistent range of O_2_:C = 0.9–1.15 mol O_2_ per mol C across all depositional phases (Fig. 2), including in the so-called Ghost Interval (GI; 7.5–2.3 cal kyr (refs. ^24,26^)). This consistency indicates that the redox characteristics of the preserved peat (O_2_:C) remained resilient even during periods of enhanced organic matter degradation associated with major hydroclimatic shifts^24,27^. Subtle but systematic variations in O_2_:C nevertheless delineate three distinct phases of peat growth, each corresponding to changes in waterlogging intensity (Fig. 2). This time-resolved record enabled us to reconstruct the basin-wide oxygen surplus, revealing a highly dynamic carbon–oxygen coupling between carbon burial and net oxygen production through the Holocene.

## Temporally resolved basin-wide O–C coupling

To quantify the ecosystem-scale redox balance, we converted time-resolved O_2_:C and TOC contents into basin-wide O_2_ equivalents (O_2_ eq) for each stratigraphic interval, with uncertainties constrained through Monte Carlo simulations (Methods). Peat accumulation initiated at ~10.6 kyr BP in response to increased precipitation^24,26,27^, generated a sustained redox imbalance of 10–30 Tg O_2_ eq yr⁻^1^ that persisted for ~2 kyr (Fig. 2 and Supplementary Fig. 1). A progressive mid-Holocene drying trend^24,28^ triggered an abrupt 80% decline in net C accumulation within centuries. During the Ghost Interval, the O_2_ surplus diminished to 0.5–5 Tg O_2_ eq yr^−^^1^ reflecting a system approaching redox balance, that is, where photosynthetic O_2_ production was nearly perfectly offset by respiratory consumption (equation (1)). In contrast, the most recent peat interval (2.3 cal kyr BP) records a renewed pulse of peat growth, with O_2_ eq fluxes rebounding to 20–45 Tg yr⁻^1^ (Supplementary Section 2). Although these changes contributed negligibly to the global atmospheric O_2_ reservoir^29^—the product of over 2.3 billion years of accumulation^30^—the O_2_ eq values provide a measure of the oxygen effectively released per mole of C escaping reoxidation. The importance therefore lies not in absolute O_2_ accumulation but in the dynamic switching of the peatland system between contrasting redox regimes. We therefore interpret these tropical peatlands as hydroclimate-sensitive ‘C–O_2_ valves’.

**Fig. 3:**
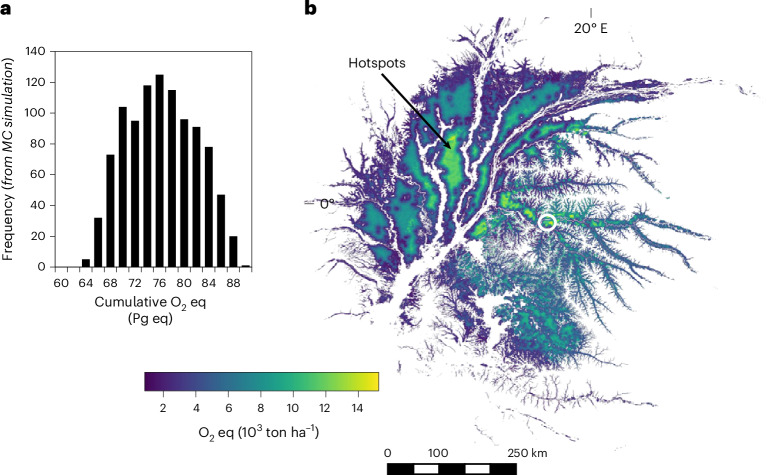
Cumulative O_2_ production derived from core IMB100 and Monte Carlo simulation. **a**, The total O_2_ production over the entire peat deposit is 76 ± 10 (2σ) Pg for the uniform sample of peat density between 0.15 and 0.19 g cm^−1^ (refs. ^22^^,23^), basin area uniformly sampled between 167,600 km^2^ (true peatland map) and 185,900 km^2^ (peat thickness and carbon density map)^23^ and uncertainties of ±10% on redox capacity values. **b**, Map of integrated O_2_ production (ton ha^−1^) in the central Congo Basin. Yellowish colour highlights hotspots (>11 Gg O_2_ eq ha^−^^1^), primarily deep peatlands (>2 m), driving 51% of total output. The white circle indicates location of IMB100 core.

Integrating these flux pulses over the Holocene, using high-resolution geospatial carbon-stock mapping (Fig. 1) yields a cumulative redox imbalance equivalent to 83 [68–100] Pg O_2_ eq across the Congo Basin (Fig. 3). This long-term surplus reflects sustained net C burial throughout the basin’s development. Notably, deep peat deposits (>2 m), although covering ~30% of the total peatland area, account for >50% of this cumulative flux (Fig. 3).

Could peatlands worldwide rival the Congo Basin? Assuming that the O_2_:C stoichiometry measured here (0.9–1.15) is broadly representative of peat, supported by its consistency both preserved and highly degraded intervals (Fig. 2), we estimate that tropical peatlands contributed 27 [22–33] Tg O_2_ eq yr^−^^1^ over the Holocene, with boreal systems adding a further 109 [93–125] Tg O_2_ eq yr^−^^1^ (Table 1 and Supplementary Section 11), which combines to a total of 136 [115–158] Tg O_2_ eq yr⁻^1^. Although modest compared to global gross primary productivity, this flux is substantial when compared to major long-term sinks in the coupled C–O_2_ cycle. Oxidative weathering of fossil organic carbon and continental sulfide minerals is estimated to consume ~106–267 Tg O_2_ eq yr^−^^1^ and ~39–78 Tg O_2_ eq yr^−^^1^, respectively (Supplementary Sections 12 and 13). Our results therefore suggest that global peatlands may have offset 40–95% of continental oxidative weathering sinks during the Holocene, with the Congo Basin alone accounting for 4–7% of this total.

## Threshold dynamics and excitable ecosystem response

The Congo peatland’s strikingly rapid, centennial-scale response to hydroclimatic perturbations reveals a nonlinear, threshold-driven behaviour that contrasts sharply with the smoothed, millennial-scale flux variability characteristic of the marine carbon cycle (~10^4^ years (ref. ^31^)). Our record shows that gradual changes in regional hydroclimate, well documented by long-term trends in leaf-wax δD^24,26^ and independent palaeotemperature reconstructions^32^, were amplified into a basin-wide ecosystem reorganization. This pattern is consistent with an excitable system response, in which slow, continuous forcings trigger an abrupt, large-amplitude shift once a critical threshold is crossed^31^. During the Ghost Interval alone, this transition resulted in a net missed sequestration of 44–53 Pg C, (Supplementary Section 13). The Ghost Interval can be viewed as a forcing on centennial timescales — with perturbation rate and mass within one order of magnitude of Rothman’s critical threshold (ref. ^33^) of carbon cycle disruptions (Supplementary Section 14). Viewed on millennial to longer timescales, however, this event is not a net forcing but a restorative one: it transiently restores redox balance, as enhanced respiratory flux temporarily offsets photosynthetic CO_2_ fixation (Fig. 2)^33^. Although regionally confined and temporally brief, this episode demonstrates that tropical peatlands can function as high-frequency, climate-sensitive valve within the global carbon–oxygen system. Accounting for such fast terrestrial pulses nested within the slower marine-dominated cycle is crucial for resolving the dynamics of Earth’s long-term oxygen–carbon coupling, both in the past and under future projected climate change.

## Geological analogue and future perspectives

The comparatively young basal age of most modern and active peatlands (typically <40–100 kyr (refs. ^10,34^) may obscure their deeper geological significance. Yet, peat formation is not a recent phenomenon. The existence of ancient peat ecosystems far more extensive and long-lived than those of today suggests that similar ‘redox valve’ dynamics may have once operated at continental scales and over geological timescales. Compelling examples include Miocene lignites in Australia and Borneo, which exceed 200 m in thickness^35,36^; Cretaceous coal deposits spanning western North America from the Arctic to Mexico^37^ and Carboniferous coal from North America and Europe that record tens of millions of years of C accumulation in peat-forming swamps^38^. With the rise of a terrestrial biosphere in the early Phanerozoic, these ecosystems created novel terrestrial carbon reservoirs that are considered responsible for driving major shifts in atmospheric composition^8^. Within this broader context, our findings provide a key mechanistic insight: tropical peatlands are capable of generating O_2_ surpluses at rates comparable to those attributed to marine organic–carbon burial^6,39^ but with substantially greater temporal variability and sensitivity to hydroclimate forcing.

The threshold behaviour observed in the Congo peatland presents many features of a canonical slow-fast dynamic, and warrants formal dynamic analysis. The apparent timescale separation probably stems from the contrast between slow autotrophic peat accumulation (10^2^–10^3^ yr) and rapid hydrological and microbial responses (10^0^–10^1^ yr) to sustained climate forcing^24,26,28^.

Another key next step is to explore the broader applicability of these dynamics beyond the Congo Basin. The variable O_2_:C stoichiometry documented in this study (Fig. 2) underpins our global extrapolation, yet it opens an exciting opportunity to test whether this behaviour is a universal peatland trait or reflects distinctive tropical conditions. Applying the same high-resolution combustion redox-titration method to boreal and temperate peatlands, where glacial history, permafrost dynamics and fire regimes exert different controls on carbon preservation, would clarify how contrasting environmental drivers shape burial stoichiometry and threshold responses. Systematic comparisons across biomes^40–42^ will help refine global flux estimates and provide mechanistically grounded constraints for Earth-system models, strengthening representation of the terrestrial contribution to long-term oxygen–carbon coupling.

Our findings establish tropical peatlands as millennial-scale regulators of the coupled carbon–oxygen cycle that are highly sensitive to hydroclimate forcing. We demonstrated that the O_2_:C combustion ratio of the preserved peat deviate from the canonical 1:1 stoichiometry of OM burial but remains remarkably resilient through intense degradation. Throughout the Holocene, the Congo Basin functioned as a rapid-response ‘carbon–oxygen valve,’ producing a cumulative mol O_2_ eq surplus of 83 [68–100] Pg, through intermittent burial pulses that were abruptly curtailed during drier intervals. This sensitivity carries direct contemporary relevance: the Congo Basin is already experiencing a measurable drying trend, with dry season length increasing by 6.4–10.4 days per decade since the late 1980s^43^. If these trends intensify, the peatlands could be pushed beyond the hydroclimate threshold that triggered the Ghost Interval — potentially closing the C–O_2_ valve and shifting the system towards a near-zero net burial state. Methodologically, our approach circumvents isotopic ambiguities by directly quantifying burial redox balance, providing a framework applicable across climatic and geological contexts from resolving glacial–interglacial C–O_2_ dynamics in boreal peatlands to probing the redox process associated with the emergence of the Amazonian rainforest^44^ or the formation of the thick carboniferous coal sequences. More broadly, our findings demonstrate that tropical peatlands are an excitable component of the planetary carbon–oxygen system, capable of reorganizations rapid enough to rival geological fluxes on millennial timescales. Rather than a passive background to marine-dominated redox evolution, peat-forming ecosystems operate as a high-frequency modulator of Earth’s oxygen-balance, an influence more fully expressed in the extensive coal and continental archives of the Phanerozoic than in today’s comparatively thin surface peat deposits.

## Methods

### Materials and context

The IMB100 peat core (237 cm) was collected in 2017 from Ingende Territory, DRC, near the Momboyo River (Supplementary Section 4). Peat accumulation began ~10.6 kyr BP at this site. The Ghost Interval (7.5–2.3 cal kyr BP) shows slowed net accumulation^26^. Continental temperatures in Central Africa were relatively stable at about 22.5 °C from 13.5 to 11.5 thousand years ago, then gradually rose by approximately 0.3 °C per millennium to reach and remain at about 25 °C from the mid-Holocene onward^51^.

### Redox titrations

Sample combustion with CuO at 900 °C measures oxygen demand (*d*O_2_) for organic matter oxidation^19^, avoiding uncertainties in traditional proxies. Calibrated measurements yield a burial-specific O_2_:C ratio (0.9–1.15 mol O_2_ mol^−1^ C, ±5%) for flux calculations, scaled to the central Congo Basin (Supplementary Sections 1–3). This range is comparable to, or only slightly broader than, oxidative ratios of 1.04 ± 0.03 (ref. ^18^) and 1.06 ± 0.06 (ref. ^17^) reported for the global terrestrial biosphere from atmospheric O_2_/CO_2_ budgets and soil incubations^17,18^. The slightly broader range of 0.9–1.15 obtained here by direct high-temperature combustion reflects the high-resolution nature of our measurement, circumventing the aggregation effect of bulk isotopic studies and the specific preservation pattern of lignocellulosic compounds in peat after degradation during early diagenesis and the Ghost Interval degradation episode.

### Thermal analysis

Thermal and calorimetric analysis assessed preservation states of labile and refractory fractions. Rock-Eval assessed total organic carbon (Supplementary Section 6). Our TOC measurements are consistent with prior Congo Basin studies (for example, ref. ^22^).

### Spatial scaling

Two approaches estimated basin-wide net mol O_2_ eq production. Because peat depth at the location of the IMB100 core (that is, 184 cm) is similar to the mean peat depth modelled for the entire central Congo Basin (that is, 170 ± 90 cm) according to ref. ^23^, we combined IMB100 core *d*O_2_ values, basin area (based on two estimates: 167,600 km^2^—true peatland map and 185,900 km^2^—peat thickness and carbon density maps^23^) and peat density (0.15–0.19 g cm^−^^3^) with Monte Carlo simulations, which yielded 76 ± 10 Pg mol O_2_ eq. The TOC–*d*O_2_ correlation (Supplementary Section 6) combined with basin-wide TOC and peat depth data (Supplementary Section 7 and refs. ^22,23^) yielded 86.8 [76.6–97] Pg mol O_2_ eq. Both estimates combined yielded 83 [68–100] Pg mol O_2_ eq.

### Limitations

Our measurements refer to dry mass after sampling, and, therefore, a minor contribution from hydrocarbons dissolved in interstitial fluids, that is, mobile and highly labile, cannot be excluded for the pristine material. Sample heterogeneity, introducing ±5–10% uncertainties in *d*O_2_ measurements, is addressed via Monte Carlo simulations. Minor reducing sulfur or iron present in the samples is accounted for in the *d*O_2_ measurements, enabling scalability to more sulfidic/ferruginous marine or terrestrial sediments^19^.

## Online content

Any methods, additional references, Nature Portfolio reporting summaries, source data, extended data, supplementary information, acknowledgements, peer review information; details of author contributions and competing interests; and statements of data and code availability are available at 10.1038/s41561-026-02031-z.

## Supplementary information

Supplementary Information Supplementary Figs. 1 and 2, Table 1 and Sections 1–15.

## Data Availability

The data that support the findings of this study are available via Zenodo at 10.5281/zenodo.20081073 (ref. ^52^).

## Code availability

Codes for the geospatial computing of O_2_ production in the central Congo Basin peatlands region using GIS are available in the IRD DataSuds repository at 10.23708/8CNH4U.
