# Supplementary material for: Hydroclimate controls Congo peatland net oxygen release over the past 10,600 years
Source: Nat Geosci. 2026 Jul 10;19(8):945–50. doi: 10.1038/s41561-026-02031-z (PMC13447085; doi:10.1038/s41561-026-02031-z)
Supplement: Supplementary file 1 [file 41561_2026_2031_MOESM1_ESM.pdf]

nature geoscience

Supplementary information

https://doi.org/10.1038/s41561-026-02031-z

Hydroclimate controls Congo peatland net oxygen release over the past 10,600 years

In the format provided by the authors and unedited

Supplementary Materials for

Hydroclimate controls on Congo peatland net oxygen release over the past 10,600 years

Galvez M.E. et al, 2026.

Content

A. **Methods Extensions**
- **S1.** Redox titration and oxygen demand (dO_2)
- **S2.** Redox Stoichiometry
- **S3.** Novelty of Redox Stoichiometry
- **S4.** Materials and Thermal Characteristics
- **S5.** Supplemental Table 1
- **S6.** Geospatial analysis: TOC vs dO_2 correlation
- **S7.** Geospatial analysis: TOC vs peat thickness correlation

B. **Global Context**
- **S8.** Flux uncertainty estimation (Monte Carlo simulation)
- **S9.** Amazon O_2 production estimate
- **S10.** Marine DOC O_2 sink
- **S11.** Global peatland and geological O_2 flux estimates
- **S12.** Oxidative weathering O_2 sinks *(petrogenic carbon & sulfide weathering)*
- **S13.** Synthesis of O_2 and CO_2 deficits during the Ghost Interval
- **S14.** Carbon cycle perturbation thresholds

Tables

- **SI Table 1** — Sample-level dO_2 and TOC data for the IMB100 core *(referenced in S5 & S6)*
- **SI Table 2** — O_2 consumption associated with 2 main oxidative weathering processes: oxidation of petrogenic carbon, and oxidation of sulfides. *(referenced in S12)*

Figures

- **SI Fig. 1** — TGA kinetograms *(referenced in S4)*
- **SI Fig 2** — Relationship between TOC and dO_2, with prediction intervals.
- **SI Fig. 3** — Peat thickness vs TOC regression for the central Congo Basin *(referenced in S7)*

A. Methods Extensions

S1. Redox titration and oxygen demand for combustion dO_2

Redox titrations quantified the oxygen demand (dO_2) for sample combustion, reflecting the O_2 preserved by carbon burial. The method closely follows the protocol outlined in [1]. Briefly, after sieving (250 μm) and homogenizing the freeze-dried peat material, an aliquot is placed in a quartz tube enclosing a container filled with a large excess of CuO— the oxygen donor. The reactor is then flame-sealed under vacuum (ca. 5 μbar) and gradually heated to 900 °C where it is left to react for 10 hours. At these nominal experimental conditions, the CuO-Cu_2O redox couple buffers the partial pressure of O_2 in the system [2] at pO_2 ~10^{-2} bar within minutes[3]. These conditions are sufficient to oxidize most redox active elements present in the sample, in particular C, S and Fe [1]. A sample is considered fully oxidized when all of its elements have reached their highest oxidation state, C^{4+}, Fe^{3+} and S^{6+}. Weighing the O donor (CuO) prior to, and after, combustion provides a robust quantification of O consumption.

This weight loss, corresponding to total mass of O consumed (nO mol), provides the absolute redox capacities, ΔO_2 = ½ nO(mol), and mass specific redox capacities, $dO_2 = \Delta O_2$ (mol)/m(g), where m is the mass of the sample. Precision measured by repeated measurements of known standards is typically within 5% [1].

The metric dO_2* is obtained by normalizing the absolute redox capacity ΔO_2 to TOC (wt%) ($dO_2^* = 100 \times dO_2$ /TOC). It is therefore expressed in mol O_2/g TOC. Mass of O_2 reservoirs is provided in Pg (≡Gt), or Tg (≡Mt).

S2. Redox Stoichiometry (or combustion ratio)

The combustion ratio O_2:C (0.9–1.15 mol O_2/mol C) was calculated as:

$O_2:C = 12 \times dO_2^*$

where dO_2* is in mol O_2/g TOC (0.08–0.095), multiplied by the molar mass of C (M_C = 12 g/mol). This yields:

$O_2:C \approx 0.9–1.15$

This ratio reflects peat's composition (e.g., degraded cellulose, lignins), changing the canonical O_2 demand compared to pure carbon (O_2:C = 1). Unlike photosynthetic O_2:C ratios (~1 for glucose), this method captures burial-specific stoichiometry, a novel contribution to atmospheric O_2 modeling.

S3. Novelty of Redox Stoichiometry

The redox stoichiometry is a novel metric, distinct from photosynthetic or biomass-based ratios, as it quantifies O_2 spared by burial[3]. Unlike carbon-focused studies [4], this approach directly links terrestrial redox processes to atmospheric O_2, offering new constraints for biogeochemical models (e.g., GEOCARBSULF[5]).

S4. Materials and Thermal characteristics

The IMB100 peat core. The Imbonga (IMB100) peat core was recovered in 2017 from the Ingende Territory (Equateur Province) in the Democratic Republic of Congo (DRC) at the northern edge of a small basin filled with peat, around 800 m away from the Momboyo River, a tributary of the Ruki River (0.856693°S and 19.808078°E, 320 m; Fig. 1A). The sedimentology, age/depth model (^{14}C), and palaeo-environmental context inherent to core IMB100 are described extensively in [6]. In summary, a total of 17 fine fraction samples (<150 μm) were used to construct the age-depth model using rbacon (version 2.7) in R (version 4.1.0) [7] . 14C dates were calibrated using a mixed curve 50%:50% of north-south air-mass mixing derived from both the Northern Hemisphere curve (IntCal20, [8]) and the Southern Hemisphere curve (SHCal20, [9]).

The 237 cm-long IMB100 core is characterized by distinct phases of peat accumulation, initially transitioning from an organic carbon-rich floodplain to a forested marshland, which later became

disconnected from the river, and finally to a closed-canopy swamp forest. Peat development began at ~10.6 kyr BP in response to increasing precipitation as inferred from plant wax hydrogen isotope (δDn-C29) data [6]. By ~8.7 kyr BP, the terrestrialization of the peatland coincided with the establishment of a closed canopy forest. Before ~10.6 kyr BP, old (of up to 38 kyr BP) and scattered radiocarbon (^{14}C) ages suggest that allochthonous material was incorporated into the fluvial sediments making the chronology uncertain [6]. Between 124 cm and 100 cm (i.e. 7.5 kyr - 2.3 kyr BP), the slope of the modelled age/depth profile is 6 to 10 times lower than in the peat sections immediately below and above. This interval has been referred as the "Ghost Interval", an interval of extensively decomposed peat material observed across the central Congo peatlands [10]. A spatial analysis of radiocarbon dates across the CCB revealed that only a small proportion of the peat in the basin today began forming prior to or during the Last Glacial Maximum (LGM), with most of it appearing to have accumulated during the Holocene [11].

Thermal characteristics. The thermal and calorimetric properties of the samples were measured using a hybridized TGA/DSC-microGC system at the University of Lausanne (UNIL). The TGA/DSC3+ (Mettler Toledo) is coupled to a 990 microGC (Agilent) via a transfer line maintained at 80 °C. Samples were heated at 10 °C/min from 100 to 1000 °C under a 10:1 N_2-O_2 atmosphere. All gases used were of high purity (> 5.8) and supplied by Carbagas and Linde. Thermograms were processed using a kinetic inversion code developed by Hemingway et al. [12], adapted for the inhouse analytical setup. By converting the thermograms into kinetograms, the inversion provides apparent activation energies (aEa), assuming a constant prefactor in the Arrhenius model. We found that a prefactor of $10^{8.5}$ which reliably reproduces the known activation energy of calcite (170-190 kJ/mol) and graphite (180-190 kJ/mol) using a Model-Free Kinetics (MFK) approach[13]. All kinetograms presented here were obtained with a smoothing factor of 4 (SI Fig. 1), which optimally balances spectral detail and smoothness.

Total organic carbon (TOC). TOC data have been obtained using Rock-Eval pyrolysis at UNIL. The TOC values were obtained on the same sample material that has been used for the dO_2 analyses (and hence for establishing the TOC vs. dO_2 correlation). The principle of Rock-Eval analysis is explained in details elsewhere [14,15]. In brief, two heating ramps are determined, the first under N2 atmosphere and the second under air. The gas produced during the first ramp (200 to 650 °C) is analyzed for hydrocarbons by a flame ionization detector (FID) and for CO_2 (and CO) using an infrared (IR) detector. The residue of the first pyrolysis step is introduced in a combustion chamber, and the evolved gas is analyzed for CO_2 (and CO) [14,15]. The total organic carbon (TOC) parameter is obtained following a standard procedure as revised by [16]. In the absence of carbonates, the organic C content corresponds to the sum of all the carbon moieties (TOC and MINC, which represent the organic and mineral C content, respectively) released during pyrolysis and oxidation. Ultimately, a correcting factor of 1.166256 is applied to biopolymer-rich samples such as forest soil litter or peat material. This correction to the 'hydrocarbon yield factor' [17] ensures compatibility between Rock-Eval TOC- (TOC-RE) and EA TOC- (TOC-EA) derived values [16]. This also ensures consistency with the basin wide TOC data published in [10,18] that are also used here for the basin wide GIS O_2 mapping.

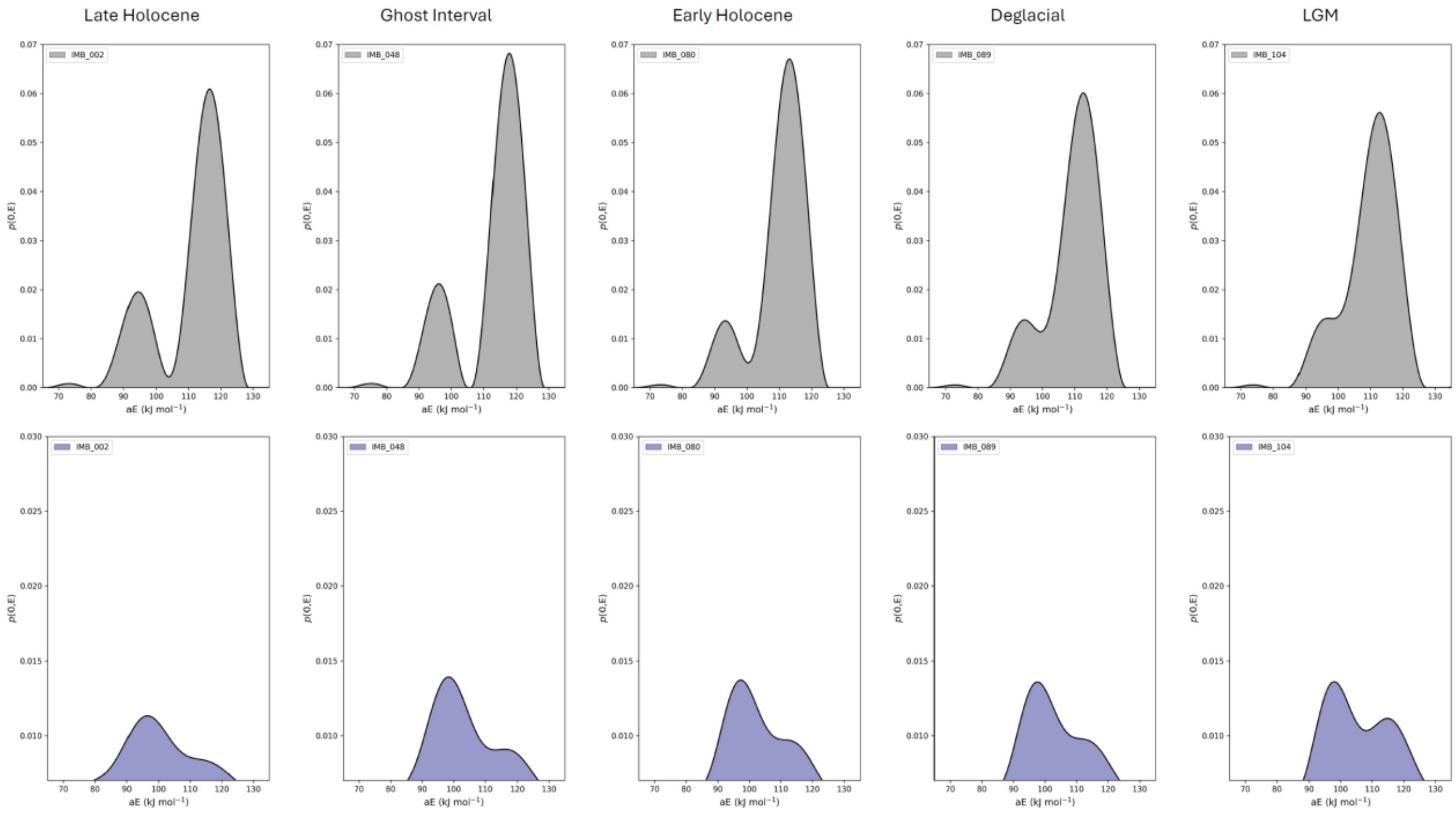

SI Fig. 1. TGA kinetograms. Sedimentary material thermal characteristics were obtained by TGA-microGC. Probability density distribution, p(o,E), are based on mass loss characteristics of the sample in oxidizing atmosphere. We identified two organic matter pools in the IMB100 core (Fig. 1A,B) using thermogravimetric analysis: a labile fraction (decomposing at 370°C, activation energy E = 90 kJ/mol), comprising fresh plant litter and carbohydrates, and a refractory fraction (450°C, E = 110 kJ/mol), including lignocellulosic and aromatic compounds. The labile-to-refractory (L/R) ratio declined from 0.3 in surface peats to 0.2 at depth, reflecting selective preservation. During the Ghost Interval, both fractions showed a 5 kJ/mol E increase, indicating drier conditions favored recalcitrant compounds.

S5.

SI Table 1: Main parameters from thermogravimetric analysis. TOC values, peak area of each labile (Peak 1) and refractory (Peak 2) fractions of the thermograms and their ratio P1/P2. Peak_1_Ea and Peak_2_Ea refer to the apparent activation energy of each peaks. The redox capacity per gram of sample is dO_2, and the redox capacity per gram of TOC is dO_2*. These ratio are used to compute the stoechiometric ratio O_2:C (cf. S1 and S2 for details).

Samples	Depth (cm)	Depth_mean (cm)	Thickness (cm)	Age med. (yr)	TOC (raw %)	TOC (corr %)	Peak_1_Ea (kJ/mol)	peak_1 area AU	Peak_2_Ea (kJ/mol)	peak_2 area AU	P1/P2 ratio	dO_2 (µmol/g)	$dO2*$ (µmol/g TOC)	Stoechiometric ratio (O_2:C mol/mol)	Time interval
IMB1.002	16-18	17	19.0	215	39.1	47.4	94.2	0.2	116.8	0.8	0.3	44021	92965	1.12	Late Holocene
IMB1.004	20-22	21	4.0	320	44.0	52.8	95.7	0.2	117.8	0.8	0.3	48271	91358	1.10	Late Holocene
IMB1.006	24-26	25	4.0	442	42.0	50.8	95.7	0.2	117.3	0.8	0.3	46832	92242	1.11	Late Holocene
IMB1.008	28-30	29	4.0	570	43.3	52.0	92.7	0.2	114.3	0.8	0.3	48998	94145	1.13	Late Holocene
IMB1.010	32-34	33	5.0	703	44.5	53.9	92.7	0.2	114.3	0.8	0.2	48114	89325	1.07	Late Holocene
IMB1.013	38-40	39	6.0	905	44.0	53.7	93.7	0.2	115.3	0.8	0.3	46111	85858	1.03	Late Holocene
IMB1.016	44-46	45	6.0	1007	41.9	50.9	95.2	0.2	116.8	0.8	0.3	46875	92121	1.11	Late Holocene
IMB1.019	50-52	51	6.0	1122	41.6	50.2	95.7	0.2	117.8	0.8	0.3	47286	94138	1.13	Late Holocene
IMB1.022	56-58	57	6.0	1240	45.8	55.5	95.7	0.2	117.3	0.8	0.3	46038	82986	1.00	Late Holocene
IMB1.025	62-64	63	5.0	1360	40.8	49.7	94.7	0.2	116.8	0.8	0.3	na	na	na	Late Holocene
IMB1.027	66-68	67	4.0	1435	42.1	51.1	94.7	0.2	116.3	0.8	0.3	47627	93282	1.12	Late Holocene
IMB1.029	70-72	71	4.0	1509	42.9	52.6	94.7	0.2	115.8	0.8	0.3	47154	89713	1.08	Late Holocene
IMB1.031	74-76	75	5.0	1572	41.9	50.8	95.2	0.2	116.8	0.8	0.3	46538	91535	1.10	Late Holocene
IMB1.034	80-82	81	6.0	1676	42.3	51.8	95.2	0.2	116.8	0.8	0.3	46767	90310	1.08	Late Holocene
IMB1.037	86-88	87	6.0	1782	41.7	50.8	95.2	0.2	116.8	0.8	0.3	46616	91824	1.10	Late Holocene
IMB1.040	92-94	93	4.0	1882	43.9	53.0	95.7	0.2	117.8	0.8	0.3	na	na	na	Late Holocene
IMB1.041	94-96	95	3.0	1917	44.6	54.1	95.2	0.2	117.3	0.8	0.3	44097	81471	0.98	Late Holocene
IMB1.043	98-100	99	3.0	2056	43.0	52.3	95.2	0.2	116.8	0.8	0.3	48117	92053	1.10	Ghost Interval
IMB1.044	100-102	101	2.0	2257	43.3	52.6	95.7	0.2	117.3	0.8	0.3	48924	92966	1.12	Ghost Interval
IMB1.045	102-104	103	2.0	2590	46.1	56.0	95.2	0.2	116.8	0.8	0.3	48473	86558	1.04	Ghost Interval
IMB1.046	104-106	105	2.0	2905	44.5	53.6	95.7	0.2	117.3	0.8	0.3	49042	91425	1.10	Ghost Interval
IMB1.047	106-108	107	2.0	3042	47.4	57.0	96.2	0.2	117.3	0.8	0.3	50205	88082	1.06	Ghost Interval
IMB1.048	108-110	109	2.0	3178	47.1	56.6	96.2	0.2	117.8	0.8	0.3	51913	91729	1.10	Ghost Interval
IMB1.049	110-112	111	3.0	3578	46.8	56.6	96.2	0.2	117.8	0.8	0.3	51313	90693	1.09	Ghost Interval
IMB1.051	114-116	115	3.0	4890	48.0	58.2	95.2	0.2	116.8	0.8	0.2	51270	88158	1.06	Ghost Interval
IMB1.052	116-118	117	2.0	5621	47.2	57.1	96.2	0.2	117.8	0.8	0.2	50465	88372	1.06	Ghost Interval
IMB1.053	118-120	119	3.0	6359	45.6	55.0	95.2	0.2	116.8	0.8	0.3	48693	88589	1.06	Ghost Interval
IMB1.055	122-124	123	4.0	7496	44.1	53.5	94.7	0.2	115.8	0.8	0.2	46984	87839	1.05	Ghost Interval
IMB1.057	126-128	127	3.0	8251	44.2	53.9	94.7	0.2	116.3	0.8	0.2	46949	87113	1.05	Early Holocene
IMB1.058	128-130	129	2.0	8439	43.2	52.6	94.2	0.2	115.8	0.8	0.2	na	na	na	Early Holocene
IMB1.059	130-132	131	3.0	8561	42.9	52.3	94.7	0.2	115.8	0.8	0.3	46858	89527	1.07	Early Holocene
IMB1.061	134-136	135	5.0	8649	42.8	52.0	94.2	0.2	115.8	0.8	0.2	45887	88280	1.06	Early Holocene
IMB1.064	140-142	141	4.0	8789	43.6	53.1	94.2	0.2	115.8	0.8	0.2	44072	82965	1.00	Early Holocene
IMB1.065	142-144	143	3.0	8837	43.1	52.3	94.7	0.2	115.8	0.8	0.3	45369	86790	1.04	Early Holocene
IMB1.067	146-148	147	3.0	8934	44.7	54.5	94.2	0.2	115.3	0.8	0.2	45036	82599	0.99	Early Holocene
IMB1.068	148-150	149	4.0	8989	44.2	54.3	94.2	0.2	115.3	0.8	0.2	44784	82542	0.99	Early Holocene
IMB1.071	154-156	155	6.0	9242	45.0	54.4	92.7	0.2	113.3	0.8	0.2	41573	76387	0.92	Early Holocene
IMB1.074	160-162	161	6.0	9571	42.3	51.6	92.7	0.2	113.3	0.8	0.2	40488	78480	0.94	Early Holocene
IMB1.077	166-168	167	6.0	9903	42.3	51.4	99.7	0.1	119.8	0.9	0.2	na	na	na	Early Holocene
IMB1.080	172-174	173	6.0	10227	42.4	51.5	93.2	0.1	113.3	0.9	0.2	42722	82945	1.00	Early Holocene
IMB1.083	178-180	179	4.0	10542	44.1	53.7	94.2	0.1	113.3	0.9	0.2	na	na	na	Early Holocene
IMB1.084	180-182	181	3.0	10711	42.5	51.5	94.2	0.1	113.8	0.8	0.2	42605	82673	0.99	Early Holocene
IMB1.086	184-186	185	5.0	11132	37.6	45.6	94.7	0.1	113.3	0.9	0.2	na	na	na	Deglacial
IMB1.089	190-192	191	4.0	18424	36.7	44.6	94.2	0.1	112.8	0.8	0.2	40221	90209	1.08	Deglacial
IMB1.090	192-194	193	3.0	19695	34.3	41.7	96.7	0.1	114.8	0.8	0.2	na	na	na	Deglacial
IMB1.092	196-198	197	5.0	21118	32.7	39.7	95.2	0.1	112.8	0.8	0.2	na	na	na	Last Glacial Maximum
IMB1.095	202-204	203	4.0	21475	26.6	32.5	96.2	0.1	112.3	0.9	0.1	27634	85086	1.02	Last Glacial Maximum
IMB1.096	204-206	205	3.0	21561	24.8	30.2	95.7	0.1	112.3	0.8	0.2	na	na	na	Last Glacial Maximum
IMB1.098	208-210	209	5.0	21762	21.6	26.4	97.2	0.1	113.3	0.9	0.1	22711	86115	1.03	Last Glacial Maximum
IMB1.101	214-216	215	4.0	22626	23.0	28.1	96.2	0.1	112.8	0.8	0.2	na	na	na	Last Glacial Maximum
IMB1.102	216-218	217	3.0	22790	21.1	25.7	96.2	0.1	113.3	0.8	0.2	na	na	na	Last Glacial Maximum
IMB1.104	220-222	221	5.0	23240	22.1	26.9	96.7	0.1	112.8	0.9	0.1	22845	84786	1.02	Last Glacial Maximum
IMB1.107	226-228	227	4.0	24337	20.3	24.7	101.3	0.2	119.8	0.8	0.2	na	na	na	Last Glacial Maximum
IMB1.108	228-230	229	3.0	24675	20.6	25.1	97.2	0.1	113.8	0.9	0.1	21618	86296	1.04	Last Glacial Maximum
IMB1.110	232-234	233	2.0	25405	18.8	23.1	96.7	0.1	112.8	0.9	0.1	na	na	na	Last Glacial Maximum

S6. Geospatial analysis: TOC vs dO_2

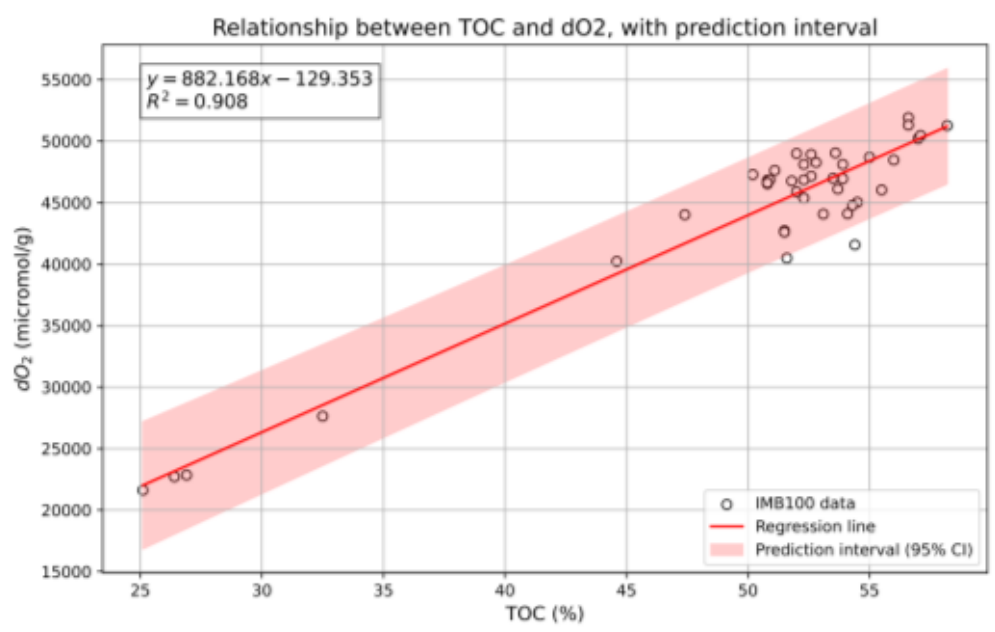

SI Fig 2: Relationship between TOC and dO_2, with prediction interval. This plot presents the correlation between TOC (corrected) values for the IMB core (this work) and dO_2 values (SI Table 1) used for O_2 production reconstructions.

S7. Geospatial analysis: Correlation between TOC and peat thickness

Using field data such as peat thickness (Fig 1B), sediment bulk density and organic carbon concentrations as measured and published in previous works [18], we have established a relationship between peat thickness and peat carbon density for the central Congo Basin (Fig. 1). In the same way, we used field data from [18] to construct a peat-thickness–total organic carbon regression (SI Fig 2) to spatially model peat TOC content (Fig. 1C). We then applied the regression between TOC values for the IMB100 core (see S6) and dO_2 values to the TOC content map to spatially model dO_2 at the basin scale (Fig. 3B).

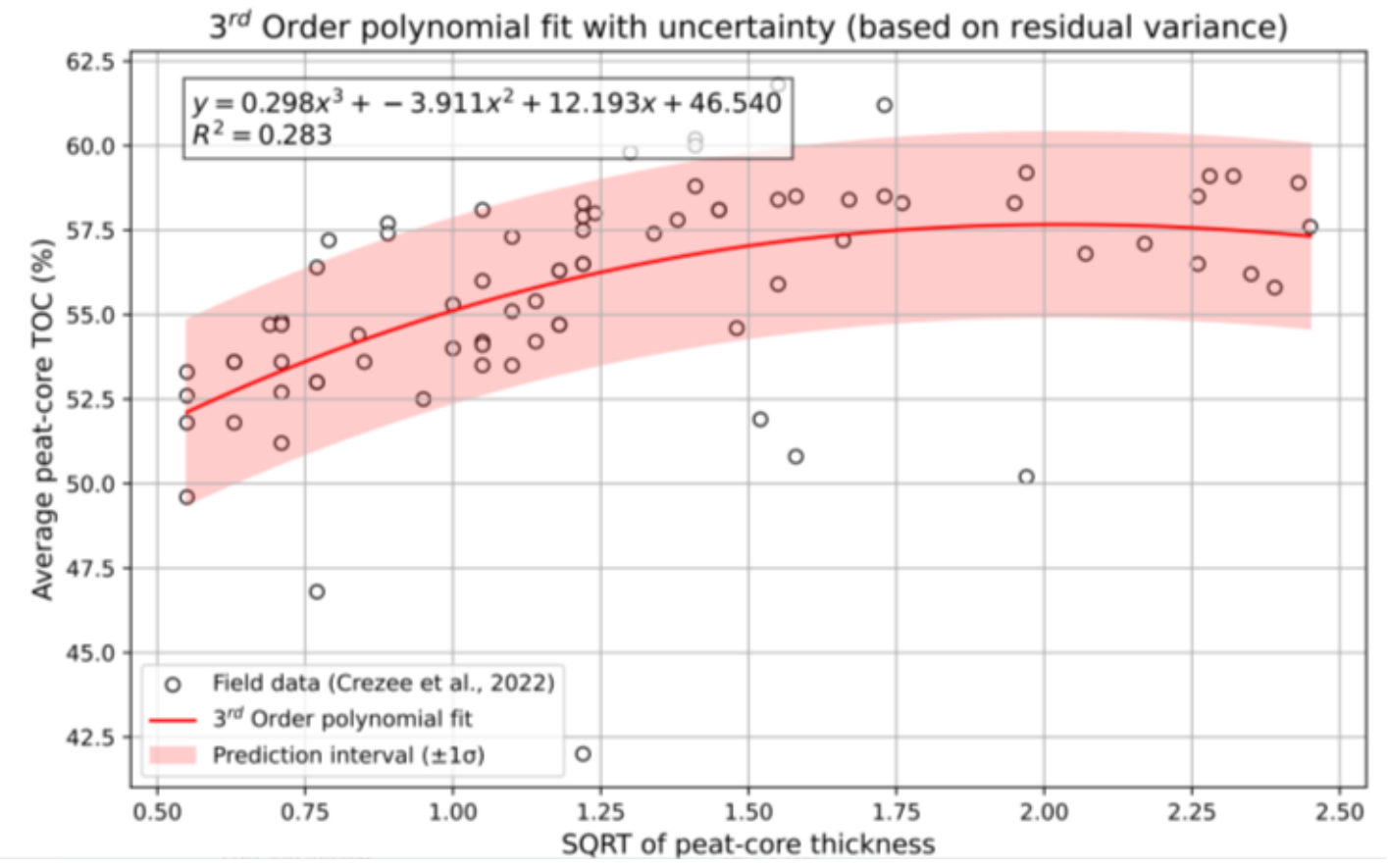

SI Fig 3. Peat thickness vs TOC regression for the central Congo Basin

S8. Flux uncertainties (±10 Pg O_2) was computed with a Monte Carlo simulation (2000 iterations) which incorporated variable peat density (normal distribution with mean 0.17 g/cm^3, 1σ=0.01 g/cm^3, providing values normally distributed in between minima at ca. 0.15 and maxima at ca. 0.19), dO_2 (±5%), and spatial variability (uniform sampling between 167600 and 185900 km^2).

B. Global Context

S9. Amazon O_2 production

We estimate the Amazon's net O_2 production at ~20–40 Tg O_2 yr^{-1}, derived from its net ecosystem production (NEP; 0.05–0.1 Pg C yr^{-1}, [19,20] and stoichiometric conversion using an O_2:C molar ratio of ~1.1 mol/mol. This estimate converts NEP carbon fluxes to O_2 production, accounting for photosynthetic O_2 release relative to carbon fixation, with uncertainties reflecting variability in NEP and stoichiometric assumptions.

S10. Marine DOC O_2 sink

The ocean holds ~662 Pg of dissolved organic carbon (DOC), 97% of which (~640 Pg) is a refractory form that persists for millennia[21,22]. This reservoir, concentrated in the deep ocean, exhibits remarkable stability with radiocarbon ages ranging from 4,000 to 6,000 years, reflecting its resistance to microbial degradation. Its vast size, comparable to atmospheric CO_2, and millennial persistence make it a critical component of long-term carbon sequestration.

S11. Global peatland and geological O_2 flux estimates

Peatlands are critical carbon sinks, contributing to atmospheric O_2 through Holocene (~11,700 years) carbon sequestration. Tropical peatlands (380,000–657,000 km^2) have accumulated ~120 [100–140] Pg C during the Holocene, including 67–92 Pg from Southeast Asian peatlands [23], 30 Pg from the Congo Basin [18], and 10–20 Pg from Amazonian/Peruvian systems [24]. Boreal peatlands (3,100,000–3,900,000 km^2) have accumulated ~480 [447–560] Pg C during the same period [25,26]. The consensus global Holocene peat C accumulation is ~600 [547–700] Pg C [25-27], representing ~30% of global soil C despite covering only 3–4% of land area. O_2 fluxes are calculated assuming an O_2:C stoichiometry of 0.9–1.15 mol/mol (mean 1.025).

Table 1 summarizes Holocene carbon stocks, accumulation rates, and O_2 fluxes, with a global total of **136 [115–158] Tg O_2 eq yr^{-1}** and a cumulative O_2 contribution of **~1,596 [1,346–1,847] Pg O_2**.

The global estimated mean Holocene oxygen production of **136 [115–158] Tg O_2 eq yr^{-1}** from peatlands represents approximately 36 % of the net marine O_2 production flux, estimated at **400 [320–480] Tg O_2 yr^{-1}** [150 [120–180] Tg C yr^{-1}]. This marine flux reflects the long-term burial of organic carbon in ocean sediments, which generates a net O_2 surplus via the imbalance in the photosynthetic-respiratory loop (Eq. 1), assuming an average O_2:C burial stoichiometry of ~1.06 mol O_2/mol C (consistent with global biosphere averages from atmospheric O_2/CO_2 budgets). The central estimate of 150 Tg C yr^{-1} (range 120–180 Tg C yr^{-1}) is a widely adopted value for modern-to-Holocene marine organic carbon burial, with continental margins (shelves, slopes, and deltas) contributing ~70–80 % of the total flux [28-31].

S12. Oxidative Weathering O_2 Sinks

- OC_{petro} weathering
  Hilton and West [31] estimate modern OC_{petro} weathering at ~70 Tg C/yr (40–100 Tg C/yr), releasing CO_2 and consuming O_2 via equation 1, main text. With a 1:1 O_2:C mol/mol ratio (2.6667 g O_2/g C):

  O_2 consumption: 70 × 2.6667 = 187 Tg O_2 yr^{-1} (107–267 Tg O_2 yr^{-1}).

- Sulfide (FeS_2) Weathering O_2 Sinks
  Burke et al. (2018) estimate modern sulfide weathering at 1.3 ± 0.2 Tmol S/yr (41.6 Tg S/yr), via
  $FeS_2 + 15/4\ O_2 + 7/2\ H_2O = FeOH_3 + 2\ H_2SO_4$
  O_2 consumption: 41.6 × 1.875 = 78 Tg O_2 yr^{-1} (66–90 Tg O_2 yr^{-1}).

SI Table 2: O_2 consumption associated with 2 main oxidative weathering processes: oxidation of petrogenic carbon, and oxidation of sulfides.

Process	C/S Flux (Tg yr^{-1})	O_2:Substrate Ratio	O_2 Consumption (Tg yr^{-1})	Source
**Petrogenic OC**	70 [40–100] C	2.67 (g O_2/g C)	187 [106–267]	Hilton & West (2020)
**Sulfide (FeS_2)**	42 [33–50] S	1.875 (g O_2/g S)	78 [66–90]	Burke et al. (2018)

Notes: Values are midpoints with ranges in parentheses. O_2 fluxes assume an O_2/C stoichiometry of 0.9–1.15 mol/mol. Global Total sums Total Tropical and Boreal contributions. Total tropical peatlands includes additional, smaller peatlands areas not reported in this table, but presented a thoroughly reviewed in {Page, 2011 #6167}. OC = Organic carbon. Ranges reflect 1σ uncertainties. Stoichiometric ratios account for complete oxidation ($OC + O_2 \rightarrow CO_2$; $FeS_2 + {}^{3}/_{4}O_2 + {}^{3}/_{2}H_2O \rightarrow FeO(OH) + 2H + 2SO_4^{2}$ (1.875:1 mass O_2:S)). 1 mol FeS_2 (120g) consumes 1.875 mol O_2 (60g). Thus 41.6 Tg S/yr × (32g S/mol)/(120g FeS_2/mol) × 1.875 × 32g O_2/mol = 78 Tg O_2/yr

S13. Synthesis of O_2 and CO_2 Deficits During the Ghost Interval

The Ghost Interval (GI; 7.5–2.3 kyr BP) caused an 80% decline in the Congo Basin's peatland O_2 flux (from 25–30 Tg O_2 yr^{-1} to 5–6 Tg O_2 yr^{-1}), which impacted both O_2 and CO_2 budgets. The 44–53 Pg C deficit represents the net missed carbon sequestration during the Ghost Interval, calculated as:

- Expected burial (high-burial mode sustained): 25–30 Tg O_2 yr^{-1} × ~6 kyr = 150–180 Pg O_2 eq
- Observed burial: 5–6 Tg O_2 yr^{-1} × ~6 kyr = 30–36 Pg O_2 eq
- O_2 deficit: 120–144 Pg O_2 eq

- Converted using the measured mean O_2:C = 1.025 (equivalent to 2.733 g O_2 per g C) → 44–53 Pg C.

This value is the carbon that was respired to CO_2 (rather than buried) relative to a scenario of continued high accumulation. It is therefore distinct from, and necessarily larger than, estimates based solely on physical peat loss from erosion or complete oxidation of previously deposited layers (~19–22 Pg C derived from the CEN-17.4 core [10]), which do not account for the much larger amount of carbon that simply never accumulated during the prolonged dry interval. The two approaches are consistent and complementary; the redox-based deficit is more comprehensive because it integrates both enhanced respiration and suppressed accumulation.

Atmospheric implication

44–53 Pg C of missed sequestration × 3.67 = 161–194 Pg CO_2. If the 44–53 Pg C of missed sequestration were instantaneously injected into the atmosphere, it would raise CO_2 by ~21–25 ppm (using 7.82 Pg CO_2 ppm^{-1}, 44–53 Pg C × 3.67 / 7.82 ≈ 20.6–24.9 ppm). However, because this shortfall occurred over ~6 kyr, the actual atmospheric anomaly would be substantially reduced by oceanic and terrestrial uptake.

S14. Carbon Cycle Perturbation Thresholds

Rothman [32] analyzed geochemical records of past mass extinction events to define critical mass and rate thresholds for catastrophic carbon-cycle destabilization. For a modern ocean inorganic carbon reservoir of ~38,000 Pg C, the critical mass is $M_c \approx 0.0082$ (normalized), equivalent to $\Delta m_c \approx 310 \pm 155$ Pg C, and the critical rate (normalized) is $r_c \approx 0.0016$ kyr^{-1}, equivalent to ~61 Pg C/kyr. The critical rate r_c indicates that carbon additions exceeding 0.16% of the ocean's carbon mass per thousand years on short timescales risk catastrophic outcomes.

It is instructive to compare these thresholds with the abrupt ghost interval carbon perturbation identified in our Congo peat record. With $\Delta m_{GI} = 44–53$ Pg C over $\tau_{env} = 6$ kyr, the ghost interval exhibits a carbon addition rate of 7.3–8.8 Pg C/kyr ($r_{GI} \approx 0.0002$). This is approximately 8-fold lower than Rothman's critical rate—and a total mass ~6–7× smaller than the critical mass threshold for abrupt events (<10000 years).

Neither condition was met, as we have shown, but these comparisons provide an intuitive grasp of the magnitude of Rothman's critical threshold itself using a system more directly accessible. Ultimately, the Ghost Interval corresponds to a transient recoupling of photosynthesis and respiration, and is therefore not a net addition to the atmosphere–ocean system capable of tipping it out of balance in Rothman's sense.

Bibliography

1 Galvez, M. E. & Jaccard, S. L. Redox capacity of rocks and sediments by high temperature chalcometric titration. *Chemical Geology* **564**, p. 120016 (2021).
2 Richet, P. *The physical basis of thermodynamics: With applications to chemistry*. (Springer Science & Business Media, 2001).
3 Galvez, M. E., Müntener, O. & Jaccard, S. L. Beyond oxygen fugacity: A compositional metric to probe Earth's redox structure. *Geophysical Research Letters* **52**, e2025GL117642 (2025).
4 Dargie, G. C. *et al.* Age, extent and carbon storage of the central Congo Basin peatland complex. *Nature* **542**, 86-90 (2017).
5 Berner, R. A. Biogeochemical cycles of carbon and sulfur and their effect on atmospheric oxygen over phanerozoic time. *Palaeogeography, Palaeoclimatology, Palaeoecology* **75**, 97-122 (1989).
6 Menges, J. *et al.* Environmental and climatic evolution of a river-proximal peatland in the Cuvette Centrale, Congo Basin. *Quaternary Science Reviews* **363**, 109445 (2025).
7 Blaauw, M. & Christen, J. A. Flexible paleoclimate age-depth models using an autoregressive gamma process. (2011).
8 Reimer, P. J. *et al.* The IntCal20 Northern Hemisphere radiocarbon age calibration curve (0–55 cal kBP). *Radiocarbon* **62**, 725-757 (2020).
9 Hogg, A. G. *et al.* SHCal20 Southern Hemisphere calibration, 0–55,000 years cal BP. *Radiocarbon* **62**, 759-778 (2020).
10 Garcin, Y. *et al.* Hydroclimatic vulnerability of peat carbon in the central Congo Basin. *Nature* **612**, 277-282 (2022).
11 Dargie, G. *et al.* Timing of peat initiation across the central Congo Basin. *Environmental Research Letters* **20**, p. 084080 (2025).
12 Hemingway, J. D., Rothman, D. H., Rosengard, S. Z. & Galy, V. V. An inverse method to relate organic carbon reactivity to isotope composition from serial oxidation. *Biogeosciences* **14**, 5099 (2017).
13 Wu, S., Jaccard, S. L. & Galvez, M. E. Technical note: Kinetically resolved volatile and redox fingerprints of geologic materials by TGA/DSC-MicroGC. *EGUsphere* **2026**, 1-25, doi:10.5194/egusphere-2026-321 (2026).
14 Lafargue, E., Marquis, F. & Pillot, D. Les applications de Rock-Eval 6 dans l'exploration et la production des hydrocarbures, et dans les études de contamination des sols. *Oil & Gas Science and Technology - Rev. IFP* **53**, 421-437 (1998).
15 Behar, F., Beaumont, V. & Penteado, H. D. B. Rock-Eval 6 technology: performances and developments. *Oil & Gas Science and Technology* **56**, 111-134 (2001).
16 Disnar, J.-R., Guillet, B., Kéravis, D., Di-Giovanni, C. & Sebag, D. Soil organic matter (SOM) characterization by Rock-Eval pyrolysis: scope and limitations. *Organic geochemistry* **34**, 327-343 (2003).
17 Delahaie, A. A. *et al.* Elemental stoichiometry and Rock-Eval® thermal stability of organic matter in French topsoils. *SOIL* **9**, 209-229, doi:10.5194/soil-9-209-2023 (2023).
18 Crezee, B. *et al.* Mapping peat thickness and carbon stocks of the central Congo Basin using field data. *Nature Geoscience* **15**, 639-644 (2022).
19 Gatti, L. V. *et al.* Amazonia as a carbon source linked to deforestation and climate change. *Nature* **595**, 388-393, doi:10.1038/s41586-021-03629-6 (2021).
20 Gloor, M. *et al.* The carbon balance of South America: a review of the status, decadal trends and main determinants. *Biogeosciences* **9**, 5407-5430 (2012).
21 Hansell, D. A., Carlson, C. A., Repeta, D. J. & Schlitzer, R. Dissolved organic matter in the ocean: A controversy stimulates new insights. *Oceanography* **22**, 202-211 (2009).
22 Hansell, D. A. Recalcitrant dissolved organic carbon fractions. *Annual Review of Marine Science* **5**, 421-445 (2013).
23 Page, S. E., Rieley, J. O. & Banks, C. J. Global and regional importance of the tropical peatland carbon pool. *Global change biology* **17**, 798-818 (2011).

24 Kelly, T. J., Lawson, I. T., Roucoux, K. H., Baker, T. R. & Coronado, E. N. H. Patterns and drivers of development in a west Amazonian peatland during the late Holocene. *Quaternary Science Reviews* **230**, 106168 (2020).
25 Yu, Z., Loisel, J., Brosseau, D. P., Beilman, D. W. & Hunt, S. J. Global peatland dynamics since the Last Glacial Maximum. *Geophysical research letters* **37**, 1021-1027 (2010).
26 Hugelius, G. *et al.* Large stocks of peatland carbon and nitrogen are vulnerable to permafrost thaw. *Proceedings of the National Academy of Sciences* **117**, 20438-20446 (2020).
27 Loisel, J. *et al.* A database and synthesis of northern peatland soil properties and Holocene carbon and nitrogen accumulation. *the Holocene* **24**, 1028-1042 (2014).
28 Berner, R. A. Burial of organic carbon and pyrite sulfur in the modern ocean: its geochemical and environmental significance. *Am. J. Sci.* **282**, 461-473 (1982).
29 Burdige, D. J. Preservation of organic matter in marine sediments: controls, mechanisms, and an imbalance in sediment organic carbon budgets? *Chemical reviews* **107**, 467-485 (2007).
30 Placitu, S. *et al.* Limited physical protection leads to high organic carbon reactivity in anoxic Baltic Sea sediments. *EGUsphere* **2025**, 1-18, doi:10.5194/egusphere-2025-3020 (2025).
31 Hilton, R. G. & West, A. J. Mountains, erosion and the carbon cycle. *Nature Reviews Earth & Environment* **1**, 284-299 (2020).
32 Rothman, D. H. Thresholds of catastrophe in the Earth system. *Science Advances* **3**, p. e1700906 (2017).
